# A pilot study of machine-learning based automated planning for primary brain tumours

**DOI:** 10.1186/s13014-021-01967-3

**Published:** 2022-01-06

**Authors:** Derek S. Tsang, Grace Tsui, Chris McIntosh, Thomas Purdie, Glenn Bauman, Hitesh Dama, Normand Laperriere, Barbara-Ann Millar, David B. Shultz, Sameera Ahmed, Mohammad Khandwala, David C. Hodgson

**Affiliations:** 1 Radiation Medicine Program, Princess Margaret Cancer Centre, University Health Network, 610 University Avenue, Toronto, ON M5G 2M9 Canada; 2 London Regional Cancer Program, London, ON Canada

**Keywords:** Machine-learning, Radiotherapy planning, Brain neoplasms

## Abstract

**Purpose:**

High-quality radiotherapy (RT) planning for children and young adults with primary brain tumours is essential to minimize the risk of late treatment effects. The feasibility of using automated machine-learning (ML) to aid RT planning in this population has not previously been studied.

**Methods and materials:**

We developed a ML model that identifies learned relationships between image features and expected dose in a training set of 95 patients with a primary brain tumour treated with focal radiotherapy to a dose of 54 Gy in 30 fractions. This ML method was then used to create predicted dose distributions for 15 previously-treated brain tumour patients across two institutions, as a testing set. Dosimetry to target volumes and organs-at-risk (OARs) were compared between the clinically-delivered (human-generated) plans versus the ML plans.

**Results:**

The ML method was able to create deliverable plans in all 15 patients in the testing set. All ML plans were generated within 30 min of initiating planning. Planning target volume coverage with 95% of the prescription dose was attained in all plans. OAR doses were similar across most structures evaluated; mean doses to brain and left temporal lobe were lower in ML plans than manual plans (mean difference to left temporal, – 2.3 Gy, *p* = 0.006; mean differences to brain, – 1.3 Gy, *p* = 0.017), whereas mean doses to right cochlea and lenses were higher in ML plans (+ 1.6–2.2 Gy, *p* < 0.05 for each).

**Conclusions:**

Use of an automated ML method to aid RT planning for children and young adults with primary brain tumours is dosimetrically feasible and can be successfully used to create high-quality 54 Gy RT plans. Further evaluation after clinical implementation is planned.

**Supplementary Information:**

The online version contains supplementary material available at 10.1186/s13014-021-01967-3.

## Introduction

Radiation therapy is an essential treatment for children and adults with brain tumours, but it can lead to important side effects including neurocognitive change, hearing loss and endocrinopathies. Designing RT treatments that maximize the likelihood of cure while minimizing side effects is crucial [1]. Although RT planning software has improved significantly in recent decades, the creation of RT plans for most tumour types is still dependent on a semi-manual iterative process of optimizing parameters to achieve an acceptable, inverse-planned RT dose distribution. This manual process of trial-and-error is operator-dependent and labor intensive, and while the resulting radiation dose distributions may meet specified clinical goals, they are not necessarily the optimal radiation plan for an individual patient. Automated planning is a method to overcome these limitations, and has been previously studied in patients with cervical cancer [2], prostate cancer [3], breast cancer [4], and lung cancer [5]. To our knowledge, no prior publication has described the successful use of automated planning to optimize radiation treatment of primary brain tumours.

In this study, we developed and evaluated an automated machine-learning RT planning method for children and adults with brain tumours. Deliverable ML-generated treatment plans were dosimetrically compared with human-generated plans that were delivered clinically.

## Materials and methods

We performed an in silico dosimetry study to evaluate feasibility of ML planning for brain tumours, and the quality of the resulting RT plans. The study was approved by the relevant institutional Research Ethics Boards.

Details of ML model development have been described previously [6–8]. In brief, an atlas of clinically-treated photon plans was first created. Within the ML pipeline, contoured structures and computed tomography (CT) imaging features were extracted by the software. Imaging features describe the appearance and texture of the imaging dataset on a per-voxel basis, and account for differences in patient anatomical geometry (see Additional file 1: supplementary materials). The first ML component used atlas regression forests (ARFs) to associate image features with observed radiation dose. This process was repeated over each voxel for the entire CT dataset, on every case in the training dataset. A second component of the ML step was designed to ensure the accuracy of dose prediction by considering contextual information to the dose-per-voxel. Since each voxel’s dose is not independent from the dose to adjacent voxels, the contextual dose links a voxel’s dose to that of nearby voxels. A conditional random field (CRF) model was used to combine these individual voxel doses and generate predicted dose distributions that were spatially accurate and realistic over anatomic regions of interest. The trained ML model predicted the dose to targets and normal tissues for a novel patient case based on the learned relationships between imaging features and per-voxel dose by automatically identifying anatomically similar training cases. The predicted dose plans are then converted into clinically deliverable single-arc volumetric arc therapy (VMAT) plans using an inverse-planning optimization algorithm that minimizes the difference between the predicted and final dose, while ensuring technical beam delivery constraints are met, to create a deliverable plan.

**Fig. 1:**
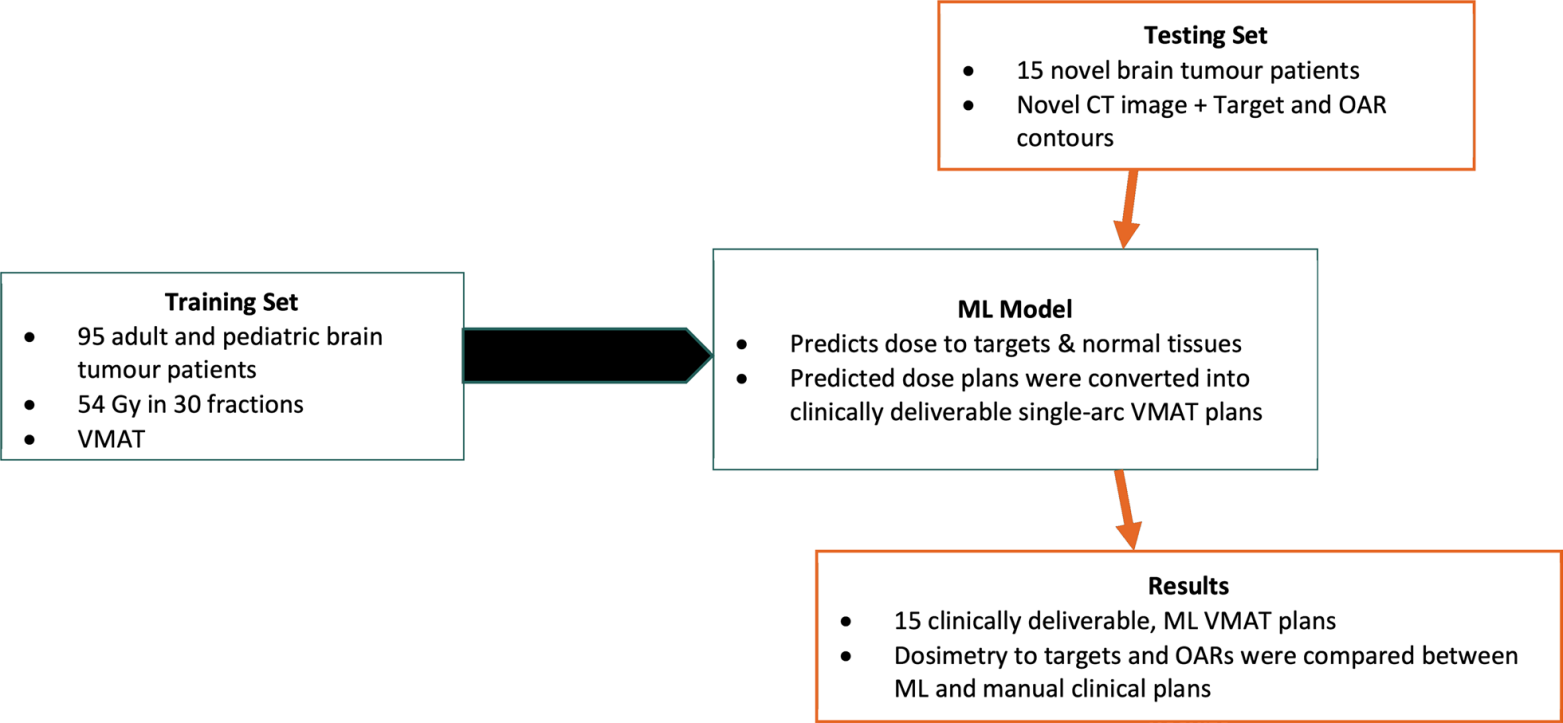
Flow diagram of study data and planning workflow

We applied this approach to a training set of 95 consecutive brain tumour patients treated from July 2016 to August 2020 at a single institution (Fig. 1). Patients receiving focal treatment (no craniospinal radiotherapy component) to 54 Gy using VMAT for an intracranial brain tumour were eligible for inclusion. RT plans met evaluation criteria listed in Table 2.

Fifteen novel brain tumour patients clinically treated with 54 Gy in 30 fractions from July 2018 to November 2020 at two institutions were then re-planned with this ML model as a testing set (Fig. 1). These patients’ novel planning CT images with target and organs-at-risk contours were input into the ML model for ML-plan generation. Dosimetry to both target volumes and OARs was reviewed and compared with the manual, human-generated plans that were delivered clinically. Target coverage, maximum doses to brainstem, optic chiasm, optic nerves, spinal cord, and mean doses to brain, hypothalamus, pituitary, cochlea, hippocampi, temporal lobes and parotids were evaluated and compared between ML and manual plans using paired t-tests.

## Results

**Fig. 2:**
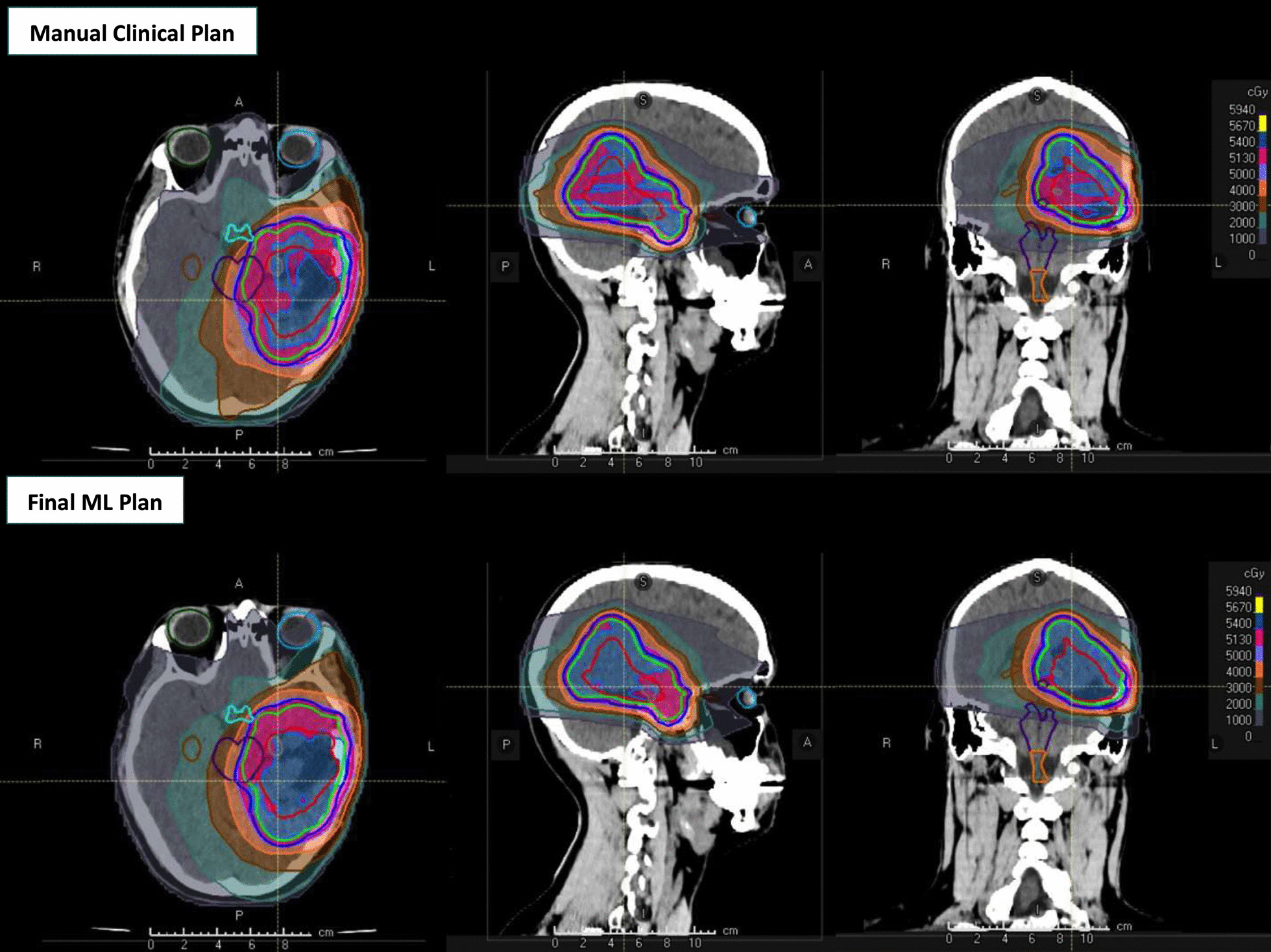
Manually-created clinical plan on top row and final ML plan bottom row respectively. Axial, sagittal and coronal views are shown from left to right. Red, green and blue lines represent the gross tumor, clinical target and planning target volumes, respectively

Details of our patient cohort are shown in Table 1. ML plans were successfully created for all 15 patients in the testing set. An example case is shown in Fig. 2, with representative manually-created clinical plan and the clinically-deliverable ML plan. All ML plans were generated within 30 min of initiating planning.

**Table 1:** Patient characteristics in training and testing set

Characteristics	Training set (n = 95)	Testing set (n = 15)
Age at RT, median (range)	24 (2, 40)	35 (13, 71)
Pediatric, age < 18 (%)	38%	13%
Female (%)	49%	33%
*Diagnosis*		
Glioma	62	9
Meningioma	6	3
Ependymoma	11	2
Medulloblastoma*	2	–
Craniopharyngioma	3	–
Others	11	1
*Tumor location*		
Supratentorial	45	10
Infratentorial	50	5
*Tumor laterality*		
Midline	45	5
Lateralized—left	18	7
Lateralized—right	32	3

*54 Gy treatment for recurrence

To evaluate ML plans in the testing set and compare with the manual plans, we first applied pre-specified plan evaluation criteria to both. The results of this comparison are shown in Table 2. Similar target coverage was observed in both ML and manual plans; at least 95% of PTV received > 51.3 Gy (95% of prescription) in all ML and manual plans. Maximum chiasm dose was < 54 Gy in 14 ML vs 15 manual plans; maximum brainstem dose was < 54 Gy in all 15 ML vs 13 manual plans.

**Table 2:** Evaluation criteria applied to manual and ML plans

Regions of interest	n	Criterion*	Criterion met		Outcome
			Manual plans	ML plans	
GTVp	15	D99 > 5130 cGy	14	14	Similar
CTVp_5400	15	D98 > 5130 cGy	15	15	Similar
PTVp_5400	15	D95 > 5130 cGy	15	15	Similar
PTVp_5400	15	Dmax < 5670 cGy	14	14	Similar
Brainstem	15	Dmax < 5400 cGy	13	15	ML better
Chiasm	15	Dmax < 5400 cGy	15	14	Manual better
External	15	Dmax < 5670 cGy	14	14	Similar
Eye_L	15	Dmax < 4500 cGy	15	15	Similar
Eye_R	15	Dmax < 4500 cGy	15	15	Similar
Lens_L	15	Dmax < 750 cGy	15	11	Manual better
Lens_R	15	Dmax < 750 cGy	15	13	Manual better
OpticNrv_L	15	Dmax < 5400 cGy	15	15	Similar
OpticNrv_R	15	Dmax < 5400 cGy	15	15	Similar
SpinalCord	15	Dmax < 5400 cGy	15	15	Similar

*Dx represents the dose to x% of the region of interest. Dmax represents the point max dose to the region of interest (i.e. a single voxel)

We subsequently compared quantitative dose metrics to OARs, shown in Table 3. Maximum doses to brainstem, chiasm, each eye and optic nerve, spinal cord, and mean doses to right temporal lobe, left cochlea, each hippocampus, hypothalamus, parotid and pituitary were not statistically different between ML and manual plans (*p* > 0.05 for each). The maximum in-patient dose was not statistically different between ML and manual plans. Mean doses to brain and left temporal lobe were lower in ML plans than manual plans (mean difference to left temporal, – 2.3 Gy, *p* = 0.006; mean differences to brain, – 1.3 Gy, *p* = 0.017), whereas mean doses to right cochlea and lenses were higher in ML plans (+ 1.6–2.2 Gy, *p* < 0.05 for each).

**Table 3:** Summary of dose differences to OARs between ML and manual plans

Organs at risk	n	Criterion	Mean values (cGy)		Dose differences*, ML – manual (cGy)				
			ML	Manual	Mean	*p* value	Median	Maximum	Minimum
Brainstem	15	Dmax < 5400 cGy	3820	3857	− 37	0.6984	8	601	− 1140
Chiasm	15	Dmax < 5400 cGy	3075	3267	− 192	0.3458	49	747	− 2051
External	15	Dmax < 5670 cGy	5599	5572	27	0.4019	45	220	− 238
Eye_L	15	Dmax < 4500 cGy	1417	1430	− 13	0.8923	26	549	− 614
Eye_R	15	Dmax < 4500 cGy	1211	1182	29	0.8388	56	988	− 927
Lens_L	15	Dmax < 750 cGy	586	369	217	**0.0188**	109	766	− 139
Lens_R	15	Dmax < 750 cGy	532	356	176	**0.0204**	177	814	− 218
OpticNrv_L	15	Dmax < 5400 cGy	2772	2701	71	0.5836	11	1130	− 890
OpticNrv_R	15	Dmax < 5400 cGy	2358	2342	16	0.8765	20	702	− 771
SpinalCord	15	Dmax < 5400 cGy	1271	1411	− 140	0.2440	− 9	104	− 1723
Brain	15	Dmean	1476	1603	− 127	**0.0172**	− 62	96	− 615
Brain_Temporal_L	15	Dmean	1758	1984	− 226	**0.0056**	− 155	69	− 854
Brain_Temporal_R	15	Dmean	1324	1389	− 65	0.3871	− 3	426	− 662
Cochlea_L	15	Dmean	1844	1555	289	0.2919	20	2850	− 2102
Cochlea_R	15	Dmean	1579	1417	162	**0.0276**	31	703	− 77
Hippocampus_L	14	Dmean	2005	2225	− 219	0.1441	− 90.5	642	− 1302
Hippocampus_R	14	Dmean	1760	1729	31	0.8081	11.5	781	− 749
Hypothalamus	15	Dmean	2214	2380	− 166	0.3638	− 121	804	− 1753
Parotid_L	15	Dmean	145	273	− 128	0.1303	− 11	33	− 1177
Parotid_R	15	Dmean	145	318	− 173	0.1051	− 14	18	− 1499
Pituitary	15	Dmean	2392	2311	81	0.5159	40	846	− 1231

Bolded text represents *p* value < 0.05

*Dose difference of an ROI is the dose in the ML plan minus dose in manual plan. Negative values indicate lower doses in the ML plan (better OAR sparing with ML plan)

## Discussion

To our knowledge, this is the first study to demonstrate the feasibility of using ML planning to create high quality, clinically deliverable RT plans for patients with primary brain tumours. ML plans were comparable with manual plans with respect to their ability to meet a priori plan evaluation criteria, including target coverage. Quantitative dosimetry to OARs was similar in both approaches, indicating that ML plans would be suitable to use and implement for clinical treatments.

Previous studies have demonstrated promising results using fully automated RT planning for sites with limited inter-patient variation in anatomy such as prostate, breast and lung cancer. McIntosh et al., demonstrated the feasibility of the voxel-based approach used here to create deliverable prostate cancer RT plans [7, 9] and Duren-Koopman et al. developed personalized, scripted tangential and arc-based RT planning for patients requiring breast plus locoregional lymph nodes [4]. Similarly, Creemers et al. demonstrate excellent dosimetric characteristics of automated VMAT plans in non-small cell lung cancer, as compared with manual plans [10]. Among primary brain tumours, although the intracranial contents are similar between patients, the variation in brain tumour configuration, and the variable impact of tumor and surgery on normal CNS anatomy poses unique challenges that the ML method was able to overcome. This contrasts with prior studies of automated planning, which have primarily been applied to anatomically homogeneous targets.

When creating ML models, using high-quality RT plans in the training model is critical so that ML output is similarly high-quality [11]. In the present study, we applied strict dosimetric criteria for inclusion in the training set to ensure high-quality plans were included in the ML model. Our study is limited to use of homogeneous dose prescriptions (54 Gy); different training sets and models are likely needed for use with two-phase plans or other prescriptions because of differing dose-constraints on OARs. Clinical implementation to ensure continued feasibility is required; this process is ongoing at our institution.

The potential of ML model lies in its the ability to reliably create high-quality treatment plans that were not dependent on the training or skill of the medical dosimetrist, as well as rapid creation of reliable RT plans. This has important potential to improve access to high quality RT in small practices or middle-income countries where planning expertise may be limited [12]. Further, rapid RT planning is especially important for patients requiring urgent commencement of RT, such as in children with symptomatic brainstem glioma.

## Conclusions

In conclusion, we developed and evaluated an automated machine-learning RT planning method for pediatric and adult brain tumour patients, and demonstrated the feasibility of rapidly generating clinically-deliverable ML plans that display consistent plan quality, as well as similar target coverage and OAR sparing as compared to human-generated plans used clinically. Clinical implementation of this ML treatment planning system is ongoing.

## Supplementary Information

**Additional file 1**. Details of our automated planning platform and machine-learning pipeline.

## Data Availability

The datasets used and analyzed during the current study are available from the corresponding author on reasonable request.

## Abbreviations

RT: Radiotherapy
ML: Machine-learning
OARs: Organs-at-risk
PTV: Planning target volume
VMAT: Volumetric arc therapy
CT: Computed tomography
ARFs: Atlas regression forests
