# Supplementary material for: A pilot study of machine-learning based automated planning for primary brain tumours
Source: Radiat Oncol. 2022 Jan 6;17:3. doi: 10.1186/s13014-021-01967-3 (PMC8734345; doi:10.1186/s13014-021-01967-3)
Supplement: Supplementary file 1 — Additional file 1. Details of our automated planning platform and machine-learning pipeline. [file 13014_2021_1967_MOESM1_ESM.docx]

**Supplementary Material**

Our automated planning platform uses a multi-patient, dose prediction approach to create high-quality RT plans based on learned relationships between image features and optimal dose distributions of anatomically similar patients (Supplementary Figure 1).


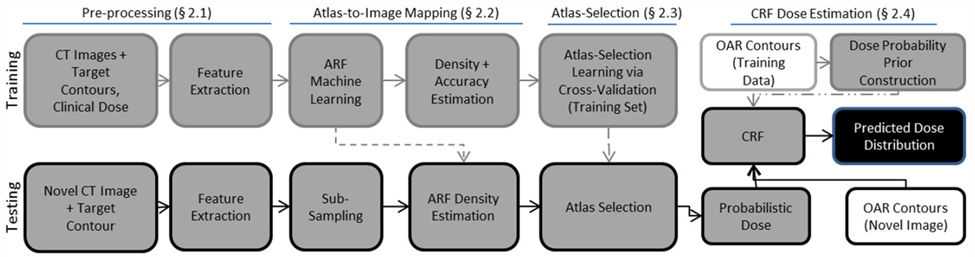


Supplementary Figure 1. Machine learning pipeline for both training and testing cases. ARF = atlas regression forest; CRF = conditional random field; OAR = organ at risk. Image derived from published work by McIntosh et al.[6]

In the ML pipeline, contoured structures and CT imaging features are extracted by the software. Imaging features describe the appearance and texture of the imaging dataset on a per-voxel basis. The first ML component uses atlas regression forests (ARFs) to model the relationship between image features (e.g. proximity to the tumor target) and radiation dose using a probability distribution function. For example, a region near the optic chiasm will have reduced probability of dose due to the need to avoid this structure. This is repeated over each voxel for the entire CT dataset in a given training case, allowing the software to learn which imaging features are important to dose prediction within each ARF. A separate ARF is trained for each training plan, consisting of the CT image, contours, and dose. A second machine-learning step is trained to predict the accuracy of an ARF given the observed features, which provides a mechanism to automatically select the most relevant ARFs for a novel patient. These most relevant ARFs provide contextual information to the dose-per-voxel. In other words, because each voxel’s dose is not independent from the dose to adjacent voxels, the contextual ARF selection links a voxel’s dose to that of nearby voxels.

This model training function is available as a standalone package (using MATLAB code) and also as part of RayStation version 8 (RaySearch Laboratories AB, Stockholm, Sweden).

The ability of a trained model to improve on clinical RT plans was then evaluated on a testing set of patients. When a new test patient is processed by the ML software platform, the patient's RT planning CT dataset is parameterized into imaging features. ARFs are evaluated on the images from the new patient and those with the highest predicted accuracy are then used in the final dose-prediction algorithm. Each voxel is assigned a predicted dose estimate using a probability distribution function. A conditional random field (CRF) model is used to combine these individual voxel doses into aggregate dose distributions that are spatially accurate and realistic, rather than assuming that the dose to each voxel is independent. After the creation of a predicted dose distribution, the final step uses inverse planning in RayStation (RaySearch Laboratories AB, Stockholm, Sweden) to create beam geometry and to design a high-quality RT plan that is clinically deliverable.

Further details are available in these works:

McIntosh C, Purdie TG. Voxel-based dose prediction with multi-patient atlas selection for automated radiotherapy treatment planning. Phys Med Biol. 2017;62:415-31.

McIntosh C, Welch M, McNiven A, Jaffray DA, Purdie TG. Fully automated treatment planning for head and neck radiotherapy using a voxel-based dose prediction and dose mimicking method. Phys Med Biol. 2017;62:5926-44.

McIntosh C, Purdie TG. Contextual Atlas Regression Forests: Multiple-Atlas-Based Automated Dose Prediction in Radiation Therapy. IEEE Trans Med Imaging. 2016;35:1000-12.
